# Oral administration of East Asian herbal medicine for peripheral neuropathy: A protocol for systematic review and meta-analysis with using association rule analysis to identify core herb pattern

**DOI:** 10.1097/MD.0000000000027644

**Published:** 2021-11-12

**Authors:** Hoseok Lee, Hee-Geun Jo, Donghun Lee

**Affiliations:** a Leehoseok Korean Medicine Clinic, 37, Gimpohangang 9-ro 76beon-gil, Gimpo-si, Gyeonggi-do, Republic of Korea; b Chung-Yeon Central Institute, 64, Sangmujungang-ro, Seo-gu, Gwangju, Republic of Korea; c Department of Herbal Pharmacology, College of Korean Medicine, Gachon University, 1342 Seongnamdae-ro, Sujeonggu, Seongnam, Republic of Korea.

**Keywords:** association rule analysis, East Asian herbal medicine, meta-analysis, peripheral neuropathy, protocol, systematic review

## Abstract

**Background:**

Peripheral neuropathy (PN) is one of the most common medical problems encountered. Since the pathophysiology and symptom manifestation characteristics of PN are very diverse, it is difficult to provide an appropriate treatment. East Asian herbal medicine (EAHM) has long contributed to the treatment of neurological and pain disorders. The goal of this systematic review is to measure the efficacy and safety of EAHM for PN and to identify core herb patterns. In order to derive a more conservative result, a random effect model will be applied regardless of the significance of heterogeneity.

**Methods:**

We will search 10 databases to identify suitable studies. There will be no restrictions regarding language or publication date. Primary outcomes will be nerve conduction velocity and response rate. Secondary outcomes will be any objective tool that can measure the efficacy of EAHM, and adverse events will be included. We will perform a meta-analysis of trials with the same intervention and outcome with comparator in a similar population. Meanwhile, in order to explore significant potential correlation in herb preparation, association rule analysis based on the Apriori algorithm will be performed on the collected composition data of herbal medicines.

**Results:**

This study will provide scientific evidence for the treatment of EAHM for PN.

**Conclusions:**

Based on the results of this review, it is expected that the efficacy and safety of EAHM for PN can be confirmed. In addition, through additional analysis using data mining techniques, it will be possible to present a core herb pattern related to this research topic.

## 1 Introduction

Peripheral neuropathy (PN) is known as one of the most common causes of patient visits to the clinic.^[1]^ The prevalence of diabetic PN or herpetic neuropathy, which are commonly observed PNs, is reported to be at least 10% to 20%.^[2,3]^ However, it is not easy to collect available PN's epidemiological data because the causes of pathology are very diverse and symptoms can occur not only in a single affected area but also in multiple nerves.^[4]^ Symptoms that may occur due to this disease include chronic pain, a decrease in nerve conduction velocity (NCV), loss of sensation, and abnormal sensations such as tingling, burning, and numbness.^[1]^ As such, PN is characterized by the fact that the pathophysiology of PN is not clear, and the symptoms of PN are not easily improved and often show a chronic course or worsen continuously.^[4]^ Thus, the medical management of this disease is challenging due to the various characteristics of PN that are difficult to manage and reduce the quality of life in patients.

Looking at the reports of several epidemiological studies, it is not difficult to see that the treatment results for PN patients are not satisfactory.^[5,6]^ The primary cause of this situation is that the accurate diagnosis and management of PN is difficult and the prognosis is poor. On the one hand, it is also a reminder that the development of more effective medications and therapeutic tools is urgently needed. East Asian herbal medicine (EAHM) deserves further investigation as a potential pharmacotherapy in this topic, given that it has long been providing benefits to patients with neurological and painful disorders in Asia.^[7,8]^ In recent years, a number of studies examining the safety and effectiveness of the use of plant preparations for neuropathy have been conducted in order to confirm the advantages of compliance with high-dose treatment, fewer side effects and safety even during long-term administration.^[9]^ In the case of East Asian medicine, the number of scientific researches verifying efficacy and safety for PN has significantly increased over the past decade.^[10,11]^ Previous systematic reviews were comprehensively dealt with the effectiveness and safety of PN, which are related to acupuncture interventions in East Asian medicine.^[12]^ However, with regard to EAHM, only a limited range of systematic reviews, associated with subcategories such as diabetic PN and chemotherapy-induced PN, have been performed.^[13,14]^

In the past decade, many randomized controlled trials (RCTs) have been conducted to assess the efficacy and safety of EAHM for PN. In addition, studies on drug discovery, which can regulate neuropathic pain based on EAHM, are also being actively conducted. Several systematic reviews^[13,14]^ associated with this topic have already been demonstrated, but unlike the case of acupuncture, a study that comprehensively reviewed the efficacy of EAHM on the overall PN caused by various causes has not yet been published. For this reason, it was difficult to derive useful pharmacological information that can be used for follow-up studies or clinical practice in the previous review. Separately, although most herbal medicine has been orally administered in East Asia, it is controversial whether different formulations such as injection or topical formulations are appropriate to be dealt with in 1 review. Therefore, the aim of the present study is to comprehensively assess the efficacy and safety of oral EAHM in the overall PN caused by multiple underlying causes. Additionally, Apriori algorithm-based association rule analysis will be performed on the various herb data to identify the core herb combination, thereby further generating useful hypotheses for subsequent drug discovery.

## 2 Methods

The present study will be conducted in accordance with the guidelines of the Cochrane Handbook for Systematic Reviews of Interventions,^[15]^ as well as the Preferred Reporting Items for Systematic Reviews and Meta-Analyses 2020 statement.^[16]^ The protocol of this systematic review was registered in PROSPERO (registration number: CRD42021252277, available from: https://www.crd.york.ac.uk/prospero/display_record.php?ID=CRD42021252277)

### 2.1 Search strategy

A comprehensive electronic search through 4 English databases (PubMed, Cochrane Library, Cumulative Index to Nursing & Allied Health Literature, Excerpta Medica database), 4 Korean databases (Korean Studies Information Service System, Research Information Service System, Oriental Medicine Advanced Searching Integrated System, Korea Citation Index), 1 Chinese database (Chinese National Knowledge Infrastructure Database), 1 Japanese database (Citation Information by NII) will be performed from inception to July 2021 by 2 investigators. The following Boolean formats adopted for the search were (mononeuropathy[MeSH] OR nerve compression syndromes[MeSH] OR neuralgia[MeSH] OR polyneuropathies [MeSH]) AND (“neuropathy”[Title/abstract] OR “peripheral neuropathy”[Title/abstract] OR “neuropathic pain”[Title/abstract] OR “neuralgia”[Title/abstract]) AND (“Medicine, Chinese Traditional”[MeSH] OR “Medicine, Kampo”[MeSH] OR “Medicine, Korean Traditional”[MeSH] OR “Herbal Medicine”[MeSH]).

### 2.2 Inclusion and exclusion criteria

#### 2.2.1 Types of studies

**Figure 1:**
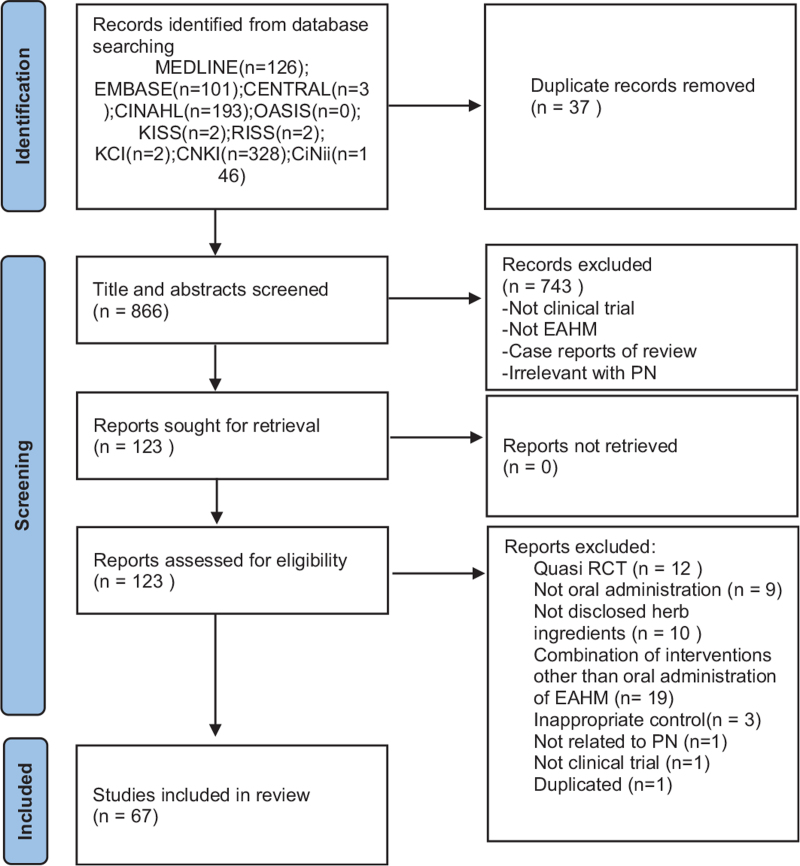
PRISMA 2020 flow diagram.

Only RCTs evaluating the efficacy and safety of oral administration of EAHM for PN will be included. In this review, we will not place restrictions on language and publication time. Some studies will be excluded if they met the following criteria: not RCT or quasi RCT; the control group is not used or is inappropriate; unrelated to PN; animal studies; review; and not published in scientific peer-reviewed journals, including postgraduate theses or dissertations. A Preferred Reporting Items for Systematic Review and Meta-Analysis 2020 flow chart will be produced to show the number of articles identified, screened, included, and excluded (shown in Fig. 1).

#### 2.2.2 Types of patients

All adults (age >18 years) diagnosed with PN will be included without restrictions on gender and nationality. The types of PN will be classified into diabetic, chemotherapy-induced, postherpetic, and other causes according to the underlying pathology.

#### 2.2.3 Types of interventions

All forms of EAHM such as decoction, granule, capsule, and a combination of EAHM and another active treatment for the management of PN will be included. The mode of delivery will be restricted to the oral intake. Studies in which East Asian medical interventions such as acupuncture, massage, or non-drug therapy will be only combined in the treatment group were excluded. Studies in which the comparators included other EAHMs will be excluded. Studies, which exemplify details of herbs constituting the revealed EAHM prescription will be also excluded.

#### 2.2.4 Types of outcome measures

Primary outcome

- NCV: improvement value of NCV measured in each part of the body.
- Response rate: rate of improvement or no improvement in symptoms such as NCV and pain, numbness, tingling, and weakness.

Secondary outcome

- Incidence rate: rate of occurrence of PN due to multiple underlying causes;
- Pain intensity: intensity of PN related to pain symptoms, as measured by instruments such as visual analogue scale or numerical rating scale;
- Toronto clinical scoring system^[17]^;
- Michigan diabetic neuropathy score^[18]^; and
- Adverse events.

### 2.3 Data extraction

Two review investigators will extract information according to the following items: 2 review investigators extracted the following items of information: first author and year of publication; type of underlying cause; patients characteristics, consisting of sample size, gender distribution, age range, and duration of disease; intervention group; control group; duration of treatment; main outcome measures and intergroup differences; adverse events; and detailed herb composition of EAHM.

### 2.4 Risk of bias in individual studies

Two review investigators independently will evaluate the risk of bias (RoB) of included studies according to the revised tool for RoB in randomized trials, RoB 2.0.^[19]^ Disagreements between the 2 review authors will be resolved by discussion. The software R version 4.1.0 was used with the robvis package to generate graphical presentations of biased risk assessments.^[20,21]^

### 2.5 Statistical analysis

#### 2.5.1 Meta-analysis

For continuous outcomes, the mean difference will be calculatedFor continuous outcomes, the mean difference will be calculated with 95% confidence interval (CI). A standardized mean difference of 95% CI will be used to express the intervention effect when the same outcome was measured using different scales. Risk ratios or odds ratios of 95% CI will be applied to represent results for dichotomous outcomes. Statistical heterogeneity across included studies will be tested using the chi-square test and I^2^ statistics. Heterogeneity will be considered statistically significant when the *P* value based on the χ^2^ test was less than .10 or I^2^ was 50% or more. If heterogeneity is identified, subgroup analysis will be performed to explore possible causes. Statistical synthesis of individual research results will be performed in the software R version 4.1.0 (R Foundation for Statistical Computing, Vienna, Austria.) using the default settings of the ‘meta’ package and the ‘metaprop’ function.^[22]^ Only the random effect model will be accepted in this review. In order to distinguish publication bias, a contour-enhanced funnel plot will be used for the outcome that included the most studies.^[23]^ For the asymmetry on the visually confirmed funnel plot, Egger test and Begg test will be additionally performed to specifically confirm the existence of publication bias.

#### 2.5.2 Association rule analysis

By analyzing the constituent herb data of EAHM collected from the included study, the potential association rules of core herb combinations will be explored. Furthermore, prior to association rule analysis, the frequency of individual herbs to be used in this analysis was checked. The R studio program (Version 1.4.1106, Integrated Development for R. RStudio, PBC, Boston, MA) will be used for the Apriori association rule analysis and plot production. A data fit will be done by using “arules” package in R studio, and the function of the R package “arulesViz” will be applied to generate plots and charts according to the results.^[24,25]^ The association rule analysis according to the Apriori algorithm is a data mining method for discovering meaningful correlations between 2 or more components included in 1 event.^[26]^ Through this, it is possible to identify the elements composing the data and the relationship between the elements, and it is being used in various types of medical research aimed at predicting the characteristics of variables.^[27–29]^ In the Apriori algorithm, support, confidence, and lift are the main metrics for measuring association. Support is a measure to evaluate the usefulness of the association rule and is the proportion of prescriptions containing a specific herb combination in the total EAHM prescription. It can be expressed as P(A∩B). Confidence indicates the likelihood that the consequent set of herbs will be included when an antecedent set of herbs is specified on an EAHM prescription. That is, support is the entire set of standard EAHM prescriptions, whereas confidence limits reference prescriptions to those that include a specific herb combination and expressed as P(A∩B)/P(A). The lift is a measure to compensate for the fact that it is not known whether the confidence is useful or a random result. The confidence of herbs A and B is divided by the confidence under the independent assumption that A does not affect B, and the formula is P(A∩B)/P(A)·P(B). When the improvement is close to 1, herb A and herb B are considered to be irrelevant because they are close to independence in probability. Conversely, if the lift value is large, the correlation is interpreted as strong. In this review, the association rules will be identified based on the minimum values for support and confidence being 20% and 80%, respectively. And among them, the core herb combination showing the most distinct association and its constituent herbs will be searched.

### 2.6 Quality of evidence according to outcome measures

The overall quality of evidence for each outcome will be evaluated according to the Grading of Recommendations Assessment, Development, and Evaluation pro.^[30]^ The Grading of Recommendations Assessment, Development, and Evaluation assessment evaluates the overall quality of evidence in 4 levels: very low, low, moderate, and high. The level of evidence is lowered according to factors such as RoB, inconsistency, indirectness, imprecision, and publication bias, respectively.

## 3 Amendments

If there is a significant modification or change of this protocol, the details and date of all amendments will be described in the final report.

## 4 Ethics and dissemination

In the process of implementing this systematic review, personal information will not be disclosed or published. This review will not infringe the rights of the subjects. Since it is not a clinical study that directly recruited subjects, ethical approval is not possible. The results of this study will be reported in a peer-reviewed scientific journal.

## 5 Discussion

This systematic review will provide comprehensive information on the efficacy and safety of EAHM for PN. The results of this review may provide benefits to patients with PN and clinicians. In addition, it is expected that the derivation of core herb pattern using data mining techniques can be used as a hypothesis worthy of follow-up research in the development of new drugs related to the subject.

## Author contributions

**Conceptualization:** Hee-Geun Jo, Donghun Lee.

**Data curation:** Hee-Geun Jo, Donghun Lee.

**Formal analysis:** Hee-Geun Jo, Hosuk Lee.

**Funding acquisition:** Donghun Lee.
